# Association between nonalcoholic fatty liver disease and extrahepatic cancers: a systematic review and meta-analysis

**DOI:** 10.1186/s12944-020-01288-6

**Published:** 2020-05-31

**Authors:** Shou-Sheng Liu, Xue-Feng Ma, Jie Zhao, Shui-Xian Du, Jie Zhang, Meng-Zhen Dong, Yong-Ning Xin

**Affiliations:** 1 Central Laboratories, Qingdao Municipal Hospital, Qingdao University, Qingdao, 266071 China; 2 Digestive Disease Key Laboratory of Qingdao, Qingdao, 266071 China; 3 Department of Infectious Disease, Qingdao Municipal Hospital, Qingdao University, Qingdao, 266011 China; 4 Departments of Nephrology, Zibo Central Hospital, Zibo, 255020 China

**Keywords:** Nonalcoholic fatty liver disease, Extrahepatic cancer, Cholangiocarcinoma, Colorectal cancer, Breast cancer, Gastric cancer

## Abstract

**Background:**

NAFLD is tightly associated with various diseases such as diabetes, cardiovascular disease, kidney disease, and cancer. Previous studies had investigated the association between NAFLD and various extrahepatic cancers, but the available data to date is not conclusive. The aim of this study was to investigate the association between NAFLD and various extrahepatic cancers comprehensively.

**Methods:**

Searches were conducted of various electronic databases (PubMed, EMBASE, Medline, and the Cochrane Library) to identify observational studies published between 1996 and January 2020 which investigated the association between NAFLD and extrahepatic cancers. The pooled OR/HR/IRR of the association between NAFLD and various extrahepatic cancers were analyzed.

**Results:**

A total of 26 studies were included to investigate the association between NAFLD and various extrahepatic cancers. As the results shown, the pooled OR values of the risk of colorectal cancer and adenomas in patients with NAFLD were 1.72 (95%CI: 1.40–2.11) and 1.37 (95%CI: 1.29–1.46), respectively. The pooled OR values of the risk of intrahepatic cholangiocarcinoma and extrahepatic cholangiocarcinoma in patients with NAFLD were 2.46 (95%CI: 1.77–3.44) and 2.24 (95%CI: 1.58–3.17), respectively. The pooled OR value of the risk of breast cancer in patients with NAFLD was 1.69 (95%CI: 1.44–1.99). In addition, NAFLD was also tightly associatied with the risk of gastric cancer, pancreatic cancer, prostate cancer, and esophageal cancer.

**Conclusions:**

NAFLD could significantly increase the development risk of colorectal adenomas and cancer, intrahepatic and extrahepatic cholangiocarcinoma, breast, gastric, pancreatic, prostate, and esophageal cancer. NAFLD could be considered as one of the influencing factors during the clinical diagnosis and treatment for the extrahepatic cancers.

## Introduction

Nonalcoholic fatty liver disease (NAFLD) has become one of the most prevalent chronic liver diseases globally. Currently, it has an estimated overall prevalence of 25.2% worldwide, and 29.62% in Asia [1–4]. The disease spectrum of NAFLD ranges from nonalcoholic fatty liver to nonalcoholic steatohepatitis (NASH), fibrosis, cirrhosis, and eventually to the hepatocellular carcinoma [5]. Considering the key physical functions of the liver, NAFLD is a complex multifactorial disease which involves sedentary life style, obesity, poor dietary habit, sarcopenia, insulin resistance, genetic susceptibility, intestinal flora and other factors [6–9]. In addition, NAFLD has been found to be closely related to many diseases such as diabetes, cardiovascular disease, and kidney disease. Thus, NAFLD is a multisystem disease with extrahepatic complications [10–16].

Accumulated evidence have shown that cardiovascular disease is the leading cause of death in patients with NAFLD, and malignancies at both gastrointestinal (liver, colon, esophagus, stomach, and pancreas) and extra-intestinal site (kidney in men, and breast in women) were also significant contributors to the mainly death of patients with NAFLD [17, 18]. Wongiarupong et al. conducted a meta-analysis to investigate the association of NAFLD with the risk of cholangiocarcinoma [19]. In their study, they found that NAFLD potentially contributes to the risk of developing cholangiocarcinoma [19]. Lee et al. also recently reported an association between NAFLD and esophageal, gastric, or colorectal cancers in Korea [20]. Mortality rates in NAFLD patients with any of these three types of cancer were markedly increased, suggesting a significant association between NAFLD and the risks of esophageal, gastric, or colorectal cancers [20].

Recently, lots of attention has been paid to the association between NAFLD and extrahepatic cancers. For example, Allen et al. have investigated the effect of NAFLD on the occurrence rate of extrahepatic cancers in a US population, and they found a nearly 2-fold increased risk of developing cancers within 21 years follow-up [21]. Due to differences in risk factors associated with various types of cancers in several countries, there is no consensus about the linkage between NAFLD and extrahepatic cancers.

The aim of this study was to identify the association between NAFLD and extrahepatic cancers comprehensively, and update the previous results of the overall association between NAFLD and extrahepatic cancers.

## Methods

### Search strategy

The multiple systematic reviews and meta-analysis of the available studies relying on the Preferred Reporting Items for Systematic Reviews and Meta-Analyses (PRISRMA) statement for the conduct of meta-analysis of observational studies were performed [22]. The following cancers were included in this study: 1) Colon neoplasm; 2) Cholangiocarcinoma; 3) Breast cancer; 4) Gastric cancer; 5) Pancreatic cancers; 6) Prostate cancer; 7) Esophageal cancer. Separate meta-analyses were performed for colon neoplasms, cholangiocarcinoma, and breast cancer. Relevant studies were identified by searching PUBMED, EMBASE, MEDLINE, and the Cochrane Systematic Review Database for studies published between 1996 and January 2020. The following search terms were used: Colon neoplasm, colon cancer, colorectal cancer, gastric neoplasm, stomach neoplasm, gastric cancers, gastric cancer, pancreatic neoplasm, pancreatic cancers, PDAC, PaC, esophageal neoplasm, esophageal cancer, breast carcinoma, breast cancer, breast neoplasm, breast tumor, breast malignant neoplasm, prostate neoplasms, prostate cancer. Only the observational studies (i.e., case-control studies and cohort studies) and written in English language were considered eligible for inclusion in this meta-analysis. Studies were excluded if they were only published as abstracts.

### Inclusion and exclusion criteria

The initially retrieved publications were reviewed by two investigators (Shou-Sheng Liu and Xue-Feng Ma) independently. Discrepancies were resolved by discussion with all investigators. Studies were included if they meet the criteria as follows: 1) studies explored the correlation between NAFLD and related cancers; 2) NAFLD or NASH was defined by either histopathological examination, imaging study or International Classification of Diseases, Ninth Revision (ICD-9) or ICD-10 codes, Hepatic steatosis index; 3) extrahepatic cancers were also defined by either histopathological examination, imaging study or ICD-9 or ICD-10 codes; 4) risk estimates (odds ratios [ORs], hazard ratios [HRs] or incidence rate ratios [IRRs]) with their corresponding 95% confidence intervals (CIs) were reported or could be calculated from the data provided; 5) studies were published full-text report in English language. The following studies were excluded: abstracts, reviews, case reports, and letters. Studies that did not provide sufficient data to calculate the risk estimates were also excluded.

### Quality assessment

Quality of the included studies was assessed independently by two authors (Shousheng Liu and Xuefeng Ma) according to the Newcastle-Ottawa Scale (NOS) [23]. The NOS is comprised of three sections: selection (up to 4 points), comparability (up to 2 points), and outcome (up to 3 points). The maximum score is 9 points. Study quality was classified as poor (score, 0–3), fair (score, 4–6), or good (score, 7–9). Discrepancies were resolved by discussion with all investigators.

### Data extraction

The following information was extracted from each study: first author, publication year, country, number of subjects, diagnosis method of NAFLD, source of patients, dates of the study, study design, diagnosis method for each cancer, adjusted confounding factors, and study quality. The data were collected independently by two investigators (Shou-Sheng Liu and Xue-Feng Ma).

### Data synthesis and analyses

Correlations between NAFLD and related cancers were calculated by OR with corresponding 95% CI. In forest plots, OR > 1 represented a risk effect, while OR < 1 represented a protective effect. Because the overall risk of extrahepatic cancers is low, HRs and IRRs in the cohort studies were similar to the ORs in the case-control studies mathematically. Thus, a combination of case-control and cohort studies was appropriate. Statistical heterogeneity among the studies was assessed according to Q and *I*^*2*^ statistics. For the Q statistic, heterogeneity was considered present when *P* < 0.1 or *I*^*2*^ > 50%. A fixed-effect model was used when literature heterogeneity did not exist; otherwise, a random-effect model was used. Publication bias was evaluated visually with funnel plots. Publication bias was considered significant when *P* < 0.05 in Begg’s test. Subgroup analyses were performed according to the design of the study. Pooled ORs were calculated by using STATA 13.0 software (Stata Corporation, College Station, TX, USA).

## Results

### Literature search and study characteristics

**Fig. 1:**
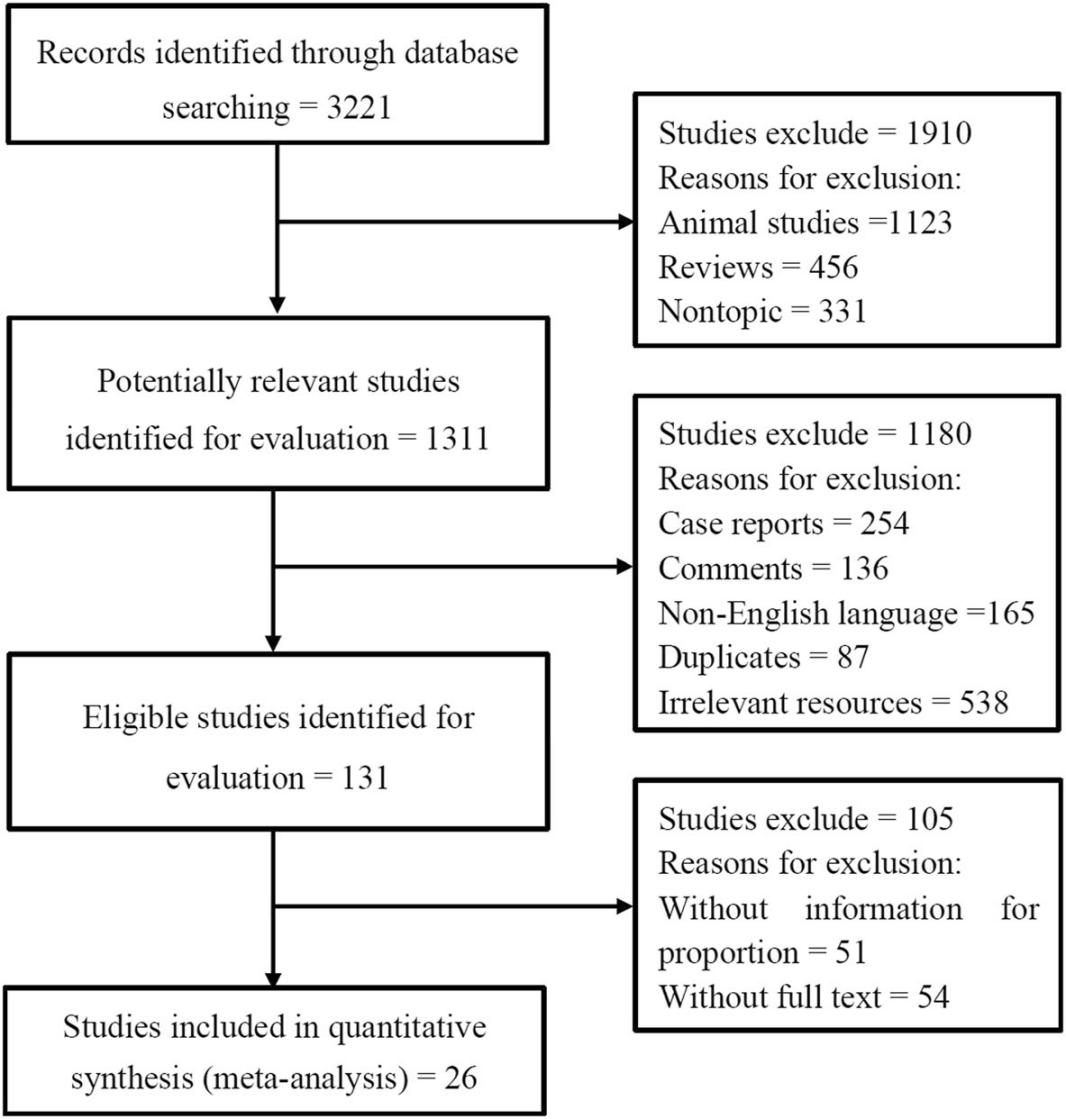
Flow chart of the literature search process conducted

A total of 3221 published studies were identified as potentially relevant from the databases searched. After removing animal studies, reviews, and nontopic studies, 1311 studies were retrieved for evaluation. After case reports, comments, non-English written articles, duplicates, and irrelevant resources were further removed, 131 studies remained for detailed evaluation. After excluding the studies which did not provide enough information of OR/HR/HRR, and those which without full text, 26 studies were included for systematic review and meta-analysis [21, 24–48] (Fig. 1). Among these selected studies, some included two or more types of the following cancers: gastrointestinal (*n* = 15), cholangiocarcinoma (*n* = 7), breast (*n* = 4), gastric (*n* = 3), pancreatic (*n* = 3), prostate (*n* = 3), and esophageal (*n* = 2).

### Association between NAFLD and gastrointestinal cancer

Fifteen of the selected studies evaluated relationships between NAFLD and risk of gastrointestinal cancers (e.g., colorectal cancer and colorectal adenoma) (Table 1). Among these studies, 7 were conducted in South Korea, 5 were conducted in China, 1 was conducted in Austria, 1 was conducted in Japan, and 1 was conducted in the USA. All of these studies were observational and included 6 cohort studies and 9 cross-sectional studies. According to NOS scores, 11 were high quality and 4 were fair quality (Table 2).

**Table 1:** Summarization of the relationship between NAFLD and all kinds of extrahepatic cancers

**Types of Cancer**	**Number of studies**	**OR/HR/IRR**	**(95%CI**)	***I***^***2***^	***P*value**
Gastrointestinal cancers					
Colorectal cancer	10	1.72	1.40–2.11	83.50%	< 0.01
Colorectal adenomas	9	1.37	1.29–1.46	49.40%	0.045
Cholangiocarcinoma					
Intrahepatic cholangiocarcinoma	6	2.46	1.77–3.44	72.60%	0.003
Extrahepatic cholangiocarcinoma	4	2.24	1.58–3.17	68.04%	0.023
Breast cancer	4	1.69	1.44–1.99	0.00%	0.727
Gastric cancer	3	1.74	1.03–2.95	73.60%	0.010
Pancreatic cancer	3	2.12	1.58–2.83	0.00%	0.476
Prostate cancer	3	1.36	1.03–1.79	81.90%	0.001
Esophageal cancer	2	1.77	1.19–2.62	0.00%	0.983

Abbreviation: *OR* odds ratio, *HR* hazard ratio, *IRR* incidence rate ratio, *CI* confidence interval

**Table 2:** Summary studies for the association between NAFLD and colorectal adenomas or colorectal cancer

**Study**	**Country**	**Sample** **size**	**NAFLD diagnosis**	**Patients source**	**Date**	**Study design**	**Colorectal cancer diagnosis**	**Adjusted confounding factors**	**Study quality**
Chen et al. 2018 [26]	China	764	Ultrasonography	Community	2014–2016	Cross-sectional	Colonoscopy	Age, sex, smoking, alcohol intake, metabolic syndrome	Good
Ahn et al. 2017 [25]	South Korea	26,540	Ultrasonography	Community	2003–2012	Cross-sectional	Histology	Age, sex, BMI, smoking, alcohol intake, first degree family history of colorectal cancer, aspirin use, fasting plasma glucose, total cholesterol, triglycerides, systolic blood pressure, use of any hypoglycemic, anti-hypertensive drugs or use of statin	Good
Chen et al. 2017 [47]	China	3686	Ultrasonography	Hospital	2007–2014	Cross-sectional	Endoscopy	Age, CEA, stage, tumor location, and tumor differentiation	Good
Pan et al. 2017 [31]	China	1793	Ultrasonography	Community	2011–2015	Cross-sectional	Colonoscopy	Age, sex, ALT, uric acid, metabolic syndrome	Good
Lee et al. 2016 [28]	South Korea	44,221	Ultrasonography	Community	2010–2011	Cross-sectional	Colonoscopy	Age, sex, BMI, smoking, family history of colorectal cancer, aspirin use, hypertension, diabetes mellitus	Good
Lin et al. 2014 [30]	China	2314	Ultrasonography	Hospital	2007–2011	Cross-sectional	Colonoscopy	Age, sex, BMI, hypertension, plasma triglycerides, uric acid, ALT, albumin, hemoglobin, platelet count	Fair
Wong et al. 2011 [36]	China	380	^1^H-MRS/Liver biopsy	Community/Hospital	2008–2010	Cross-sectional	Colonoscopy	Age, sex, BMI, smoking, family history of colorectal cancer, hypertension, diabetes mellitus	Good
Stadlmayr et al. 2011 [35]	Austria	1211	Ultrasonography	Hospital	2007–2009	Cross-sectional	Colonoscopy	Age, sex, BMI, glucose intolerance status (impaired fasting glycaemia or diabetes mellitus)	Good
Hwang et al. 2010 [34]	South Korea	2917	Ultrasonography	Hospital	2007	Cross-sectional	Colonoscopy	Age, sex, smoking, hypertension, diabetes mellitus, metabolic syndrome	Good
Allen et al. 2019 [21]	USA	276	HCIDA/ICD-9	Community	1997–2016	Cohort	ICD-9	NA	Good
Hamaguchi et al. 2019 [27]	Japan	15,926	Ultrasonography	Community	2004–2016	Cohort	Endoscopy	Sex, age and lifestyle factors including smoking habits, alcoholic consumption and physical activities and diabetes	Good
Kim et al. 2017 [24]	South Korea	NA	Ultrasonography	Community	2004–2005	Cohort	Pathology	Demographic and metabolic factors	Good
Yang et al. 2017 [32]	South Korea	882	Ultrasonography/ computed tomography	Hospital	2009–2013	Cohort	Colonoscopy	Age, sex, smoking, hypertension, diabetes mellitus, use of aspirin or lipid-lowering agents; imaging for diagnosis of NAFLD	Fair
Huang et al. 2013 [33]	South Korea	1522	Ultrasonography	Hospital	2003–2010	Cohort	Colonoscopy	Age, sex, BMI, smoking, hypertension, diabetes mellitus, metabolic syndrome	Fair
Lee et al. 2012 [29]	South Korea	5517	Ultrasonography	Hospital	2002–2006	Cohort	Colonoscopy	Age, BMI, smoking, hypertension, dyslipidemia, fasting glucose level	Fair

Abbreviation: *HICDA* Hospital International Classification of Diseases Adapted, *ICD* International Classification of Diseases

**Fig. 2:**
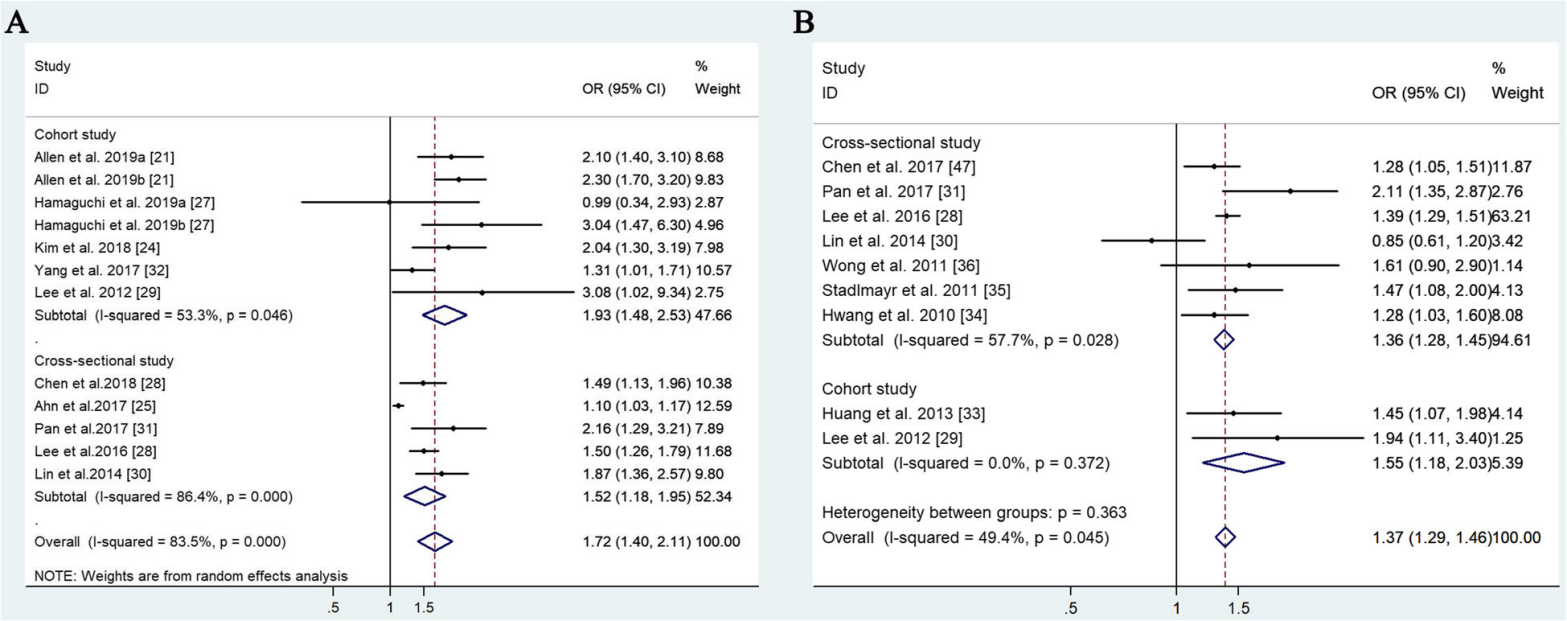
Meta-analysis of the association between nonalcoholic fatty liver disease (NAFLD) and colorectal cancer or colorectal adenomas. Forest plots are shown for colorectal cancer (**a**) and colorectal adenomas (**b**)

To investigate the association between NAFLD and the risk of colorectal cancer, pooled OR of colorectal cancer from ten studies was analyzed [21, 24–32]. A meta-analysis was conducted with the random-effect model (*P* < 0.01, *I*^*2*^ = 83.5%). The results indicated that patients with NAFLD have a significant risk of developing colorectal cancer (OR = 1.72, 95% CI: 1.40–2.11) (Fig. 2a). Publication bias was also tested by using Begg’s test. The results suggested that an obvious publication bias exists among these studies (*P* < 0.01) (Fig. 4a). Furthermore, the results of a subgroup analysis showed that the pooled OR of colorectal cancer in the cross-sectional studies [25, 26, 28, 30, 31] was 1.93 (95% CI: 1.48–2.53), and in the cohort studies [21, 24, 27, 29, 32] it was 1.52 (95% CI: 1.18–1.95) (Table 1, Fig. 2a).

To investigate the association of NAFLD and the risk of colorectal adenoma, pooled OR of colorectal adenomas from nine studies was analyzed [28–36, 47]. A meta-analysis was conducted with the random-effect effect model (*P* = 0.045, *I*^*2*^ = 49.4%). The results indicated a significant developmental risk of colorectal adenoma in patients with NAFLD (OR = 1.37, 95% CI: 1.29–1.46) (Fig. 2b). When publication bias was tested, Begg’s test indicated no obvious publication bias (*P* = 0.754) (Fig. 4b). Furthermore, the results of subgroup analysis showed that the pooled OR of colorectal adenoma in the cross-sectional studies [28, 30, 31, 34–36, 47] was 1.36 (95% CI: 1.28–1.45), and in the cohort studies [29, 33] it was 1.55 (95% CI: 1.18–2.03) (Table 1, Fig. 2b). These results suggest that patients with NAFLD have a higher risk of developing colorectal cancer and colorectal adenoma than patients without NAFLD.

### Association between NAFLD and cholangiocarcinoma

In this systematic review and meta-analysis, seven studies were included to evaluate relationships between NAFLD and the risk of cholangiocarcinomas [including intrahepatic and extrahepatic cholangiocarcinomas (ICC and ECC, respectively)] [37–43]. Three of these studies were conducted in the United States, 1 was conducted in Europe, 1 was conducted in China, 1 was conducted in Japan, and 1 was conducted in South Korea. All of these studies were cross-sectional studies. In addition, patients in four studies were from the community, while three studies were from hospital. According to NOS scores, 4 studies were high quality and 3 studies were fair quality (Table 3).

**Table 3:** Summary studies for the association between NAFLD and cholangiocarcinoma and breast cancer

**Study**	**Country**	**Sample size**	**NAFLD diagnosis**	**Patients source**	**Date**	**Study design**	**Cancer diagnosis**	**Adjusted confounding factors**	**Study quality**
**Cholangiocarcinoma**									
Petrick et al. 2017 [41]	US	328,688	ICD-9	Community	2000–2011	Case-control	ICD-9	Age, race/ethnicity, geographic region, and state buy-in status	Good
Choi et al. 2016 [38]	US	7164	Histology/Imaging	Hospital	2000–2014	Case-control	ICD-9	The differences in frequencies of aspirin current users	Good
Kinoshita et al. 2016 [39]	Japan	103	Histology	Hospital	1995–2014	Case-control	Pathology	NA	Fair
Stepien et al. 2016 [42]	Europe	495	Hepatic steatosis index	Community	1992–2000	Case-control	ICD-9	Smoking status, baseline, lifetime alcohol intake pattern, body mass index, physical activity, hepatitis B, C infection, diabetes status, CRP	Good
Lee et al. 2015 [40]	South Korea	243	Histology/Imaging	Hospital	2007–2013	Case-control	Pathology	NA	Good
Chang et al. 2013 [37]	China	25,785	ICD-9	Community	2004–2008	Case-control	ICD-9	Possible intermediate factors	Fair
Welzel et al. 2007 [43]	US	103,866	ICD-9	Community	1999–2009	Case-control	ICD-9	NA	Fair
**Breast cancer**									
Allen et al. 2019 [21]	USA	676	ICD-9	Community	1997–2016	Cohort	ICD-9	NA	Good
Kim et al. 2017 [24]	Korea	NA	Ultrasonography	Community	2004–2005	Cohort	Pathology radiology	Demographic and metabolic factors	Good
Nseir et al. 2017 [45]	Israel	146	Ultrasonography	Community	2008–2011	Cohort	Ultrasonography	NA	Good
Kwak et al. 2019 [44]	USA	540	Ultrasonography.	Community	2008–2017	Case-control	Ultrasonography	NA	Good

Abbreviation: *ICD* International Classification of Diseases

**Fig. 3:**
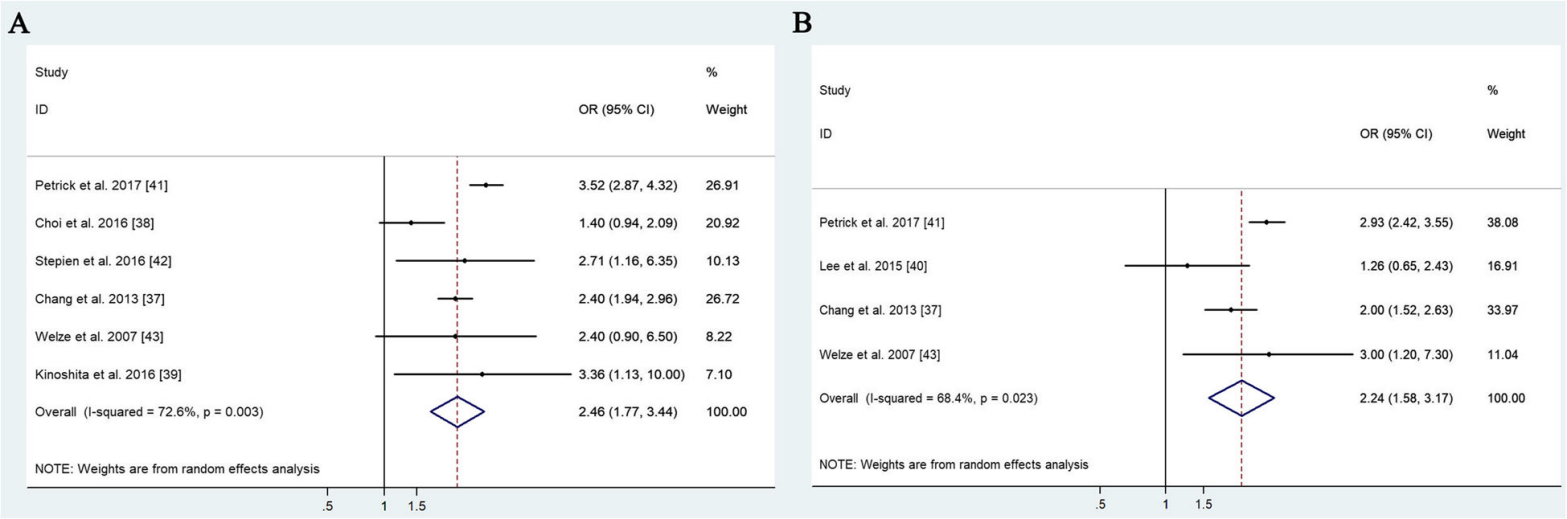
Meta-analysis of the association between nonalcoholic fatty liver disease (NAFLD) and cholangioarcinomas. Forest plots are shown for intrahepatic cholangiocarcinomas (ICC) (**a**) and extrahepatic cholangiocarcinomas (ECC) (**b**)

**Fig. 4:**
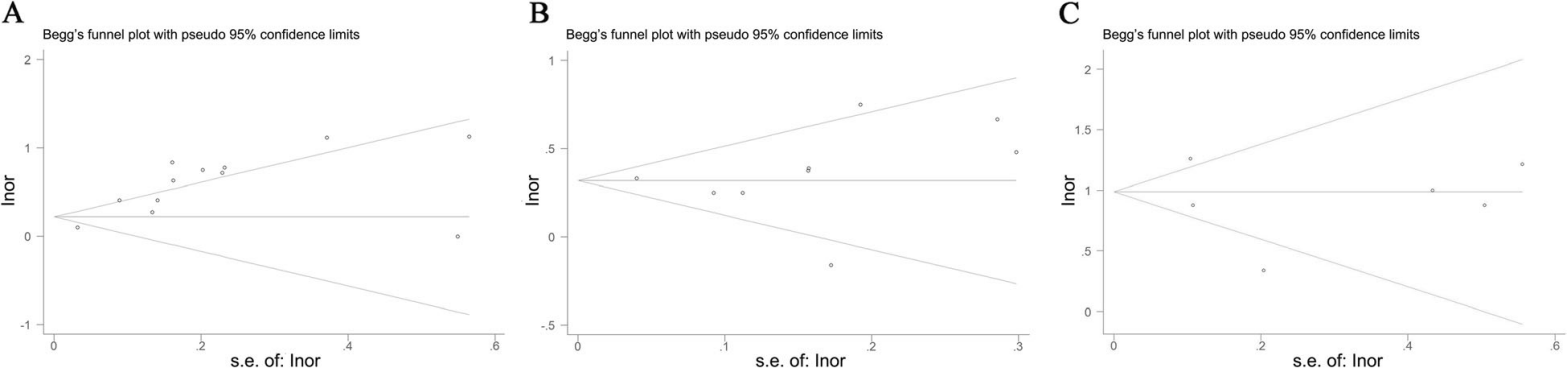
Begger’s funnel plots for publication bias are shown for colorectal cancer (**a**), colorectal adenomas (**b**), and intrahepatic cholangiocarcinomas (ICC) (**c**)

To investigate the association of NAFLD and the risk of ICC, pooled OR of ICC from six studies was analyzed [37–39, 41–43]. A meta-analysis was conducted with the random-effects model (*P* = 0.003, *I*^*2*^ = 72.60%). The results showed a significant risk of developing ICC in patients with NAFLD (OR = 2.46, 95% CI: 1.77–3.44) (Fig. 3a). In addition, according to Begg’s test, no obvious publication bias was observed among these studies (*P* = 0.501) (Table 1, Fig. 4c). To investigate the association of NAFLD and the risk of ECC, pooled OR of ECC from four studies was analyzed [37, 40, 41, 43]. A meta-analysis was conducted with the random-effect model (*P* = 0.024, *I*^*2*^ = 68.04%). The results showed that the risk of developing ECC was significantly higher in patients with NAFLD (OR = 2.24, 95% CI: 1.58–3.17) than in patients without NAFLD (Table 1, Fig. 3b). These results suggest that NAFLD may increase the risk of developing ICC and ECC.

### Association between NAFLD and breast cancer

In this systematic review and meta-analysis, four studies were included to evaluate the relationship between NAFLD and the risk of developing breast cancer [21, 24, 44, 45]. Among these studies, 2 were conducted in the United States, 1 was conducted in Korea, and 1 was conducted in Israel. Three of the studies were cohort studies, while the remaining study was a case-control study. NOS scores indicated that all four studies were high quality (Table 3). Based on the data in Table 1 and in Supplementary Fig. 1, the pooled OR of breast cancer in patients with NAFLD was 1.69 (95% CI: 1.44–1.99), which suggests that patients with NAFLD are more susceptible to breast cancer.

### Associations between NAFLD and other cancers

Three of the included studies evaluated the association between NAFLD and the risk of developing gastric cancer [21, 24, 27] (Supplementary Table 1). According to NOS scores, all three of these cohort studies were of high quality. The pooled OR of gastric cancer was 1.74 (95% CI: 1.03–2.95) (Table 1), which suggests that patients with NAFLD have a high risk of developing gastric cancer. Similarly, to investigate a possible association between NAFLD and the risk of developing pancreatic cancer, three studies were included [21, 24, 46] (Supplementary Table 1). All three cohort studies were of high quality according to their NOS scores. The pooled OR of pancreatic cancer was 2.12 (95% CI: 1.58–2.83) (Table 1), which suggests that patients with NAFLD have a high risk of developing pancreatic cancer. In addition, it was observed that patients with NAFLD have a high risk of developing prostate cancer (OR = 1.36, 95% CI: 1.03–1.79) (Tables 1 and Supplementary Table 1). Furthermore, two of the studies reported an association between NAFLD and a risk of developing esophageal cancer [21, 24], with the OR value of esophageal cancer being 1.77 (95% CI: 1.19–2.62) (Tables 1 and Supplementary Table 1).

## Discussion

NAFLD is an epidemic of chronic liver disease worldwide, and it is the manifestation of metabolic syndrome in the liver [3]. Accumulating evidence suggest that NAFLD is tightly associated with various diseases, including diabetes, cardiovascular disease, kidney disease, and cancer [11–13, 49, 50]. In recent years, more attentions have been paid to the possible association between NAFLD and the risk of certain cancers. For example, clinical observational studies have been conducted which investigate the relationship between NAFLD and the risks of developing cancer, especially extrahepatic cancers such as colon, stomach, and pancreas. Both Kim et al. and Allen et al. have conducted reviews regarding this issue [21, 24]. However, the results could not reflect the newest conclusion of the association between NAFLD and extrahepatic cancers absolutely. With the publication of new studies, a latest summary is needed to expound the new research progresses in ths issue. Therefore, the newest systematic reviews and meta-analysis were conducted to investigate the association of NAFLD with the risk of various extrahepatic cancers comprehensively in this study.

In this study, the relationship of NAFLD with gastrointestinal cancers (colorectal cancer and colorectal adenoma), cholangiocarcinomas (ICC and ECC), and other cancers (including breast, gastric, pancreatic, prostate, and esophageal) were investigated. The results obtained suggest that NAFLD is tightly associated with all of these extrahepatic cancers. However, detailed mechanism(s) to explain how NAFLD promotes tumorigenesis remain unclear. NAFLD is caused by excessive accumulation of triglycerides in the liver, which could be regarded as a type of visceral adiposity [51]. Previous reports have suggested that visceral adipose tissue may affect the function of other organs by releasing cytokines such as adipocytokines, growth factors, and some pro-inflammatory factors [52]. This hypothesis, if confirmed, could provide valuable insight into the mechanism of NAFLD in tumorigenesis.

In a previous meta-analysis which investigated the association of incident and recurrent colorectal cancer and adenoma with NAFLD, it was observed that the presence and severity of NAFLD were associated with an increased risk of incident colorectal cancer or adenomas [53]. When Mantovani et al. examined the association between NAFLD and colorectal tumors in asymptomatic adults who underwent a screening colonoscopy, they found that NAFLD was associated with a moderate increase in the risk of colorectal cancer and adenoma [54]. In the present study, both inclusion and exclusion criteria were strictly adhered to, and all suitable studies were included to investigate the association of NAFLD with the risk of developing colorectal cancer and colorectal adenoma. The results show that NAFLD significantly increases the risk of colorectal cancer (OR = 1.72, 95% CI: 1.40–2.11) and the risk of colorectal adenoma (OR = 1.37, 95% CI: 1.29–1.46) compared to healthy controls. These results are consistent with those of previous studies, and they support further investigations of the mechanism(s) by which NAFLD promotes the development of colorectal cancer and colorectal adenoma.

The associations between NAFLD and other extrahepatic cancers is less proven [55]. When Wongjarupong et al. conducted a meta-analysis to investigate a possible relationship between NAFLD and cholangiocarcinoma, they found that NAFLD was associated with both ICC (OR = 2.22, 95% CI: 1.52–3.24) and ECC (OR = 1.55, 95% CI: 1.03–2.33) [19]. In this study, the most recent publications available were added to conduct this meta-analysis [21, 24–28, 31, 32, 41, 44–48]. The results obtained show that NAFLD significantly increases the risk of developing both ICC (OR = 2.46, 95% CI: 1.77–3.44) and ECC (OR = 2.24, 95% CI: 1.58–3.17). These results are consistent with those of previous studies [19, 54]. Furthermore, links between NAFLD and breast, gastric, pancreatic, prostate, and esophageal cancers were also reviewed. Recently, Ahmed et al. reviewed the studies of extrahepatic malignancies in NAFLD systematically, but the detailed OR/HR/IRR of each cancer was not analyzed [50], therefore, this research made the well supplement and update for Ahmed’s study. Sorensen et al. have reported that patients with NAFLD in the Danish population exhibit an increased risk of lung cancer and renal cell carcinoma [55]. Similarly, Watanabe et al. have verified that NAFLD may be associated with more severe renal cell carcinoma and shorter overall survival in Japanese populations [56, 57]. However, relationships between NAFLD and lung cancer and renal cell carcinoma remain to be further studied as available data are currently insufficient. In addition, clinical studies are needed to further investigate the association between NAFLD and extrahepatic cancers. Kim et al. reported that patients with NAFLD possess the higher susceptibility to colorectal cancer in males, and breast cancer in females [24], whether there is the gender-related difference in the association of NAFLD and extrahepatic cancers remains unclear. A recent study demonstrated that patients with NAFLD are more likely to exhibit chronic inflammation with insulin resistance, which may generate a microenvironment conducive for cancer development [58, 59]. Emerging translational and epidemiologic data support that local ectopic fat may also affect functional factors, and in turn the paracrine pathway, to induce cancer development in the liver, pancreas, and breast [60, 61]. Therefore, the results of the present study are consistent with those of previous studies and they indicate that NAFLD is a risk factor for various extrahepatic cancers.

### Strength and study limitation

There were several limitations in this study. First, the tight association between NAFLD and extrahepatic cancers was investigated, but the degrees of NAFLD did not be classified. Consequently, the association of NAFLD severity with the extrahepatic cancers examined was not demonstrated. Second, a small number of studies were available to analyze the association between NAFLD and the risks of gastric, pancreatic, prostate, and esophageal cancers. Therefore, further studies are needed to focus on the risk of these extrahepatic cancers in patients with NAFLD. Third, many of the studies examined were conducted in East Asia. However, the ethnicity of the subjects in each study were not defined. Given that the risks of developing NAFLD and various cancers differ according to ethnicity, the influence of ethnicity on the relationship between NAFLD and extrahepatic cancers should be investigated in future studies.

## Conclusion

In summary, the systematic review and meta-analysis was conducted to comprehensively investigate associations between NAFLD and the risk of developing extrahepatic cancers. The results indicate that NAFLD can significantly increase the risk of developing colorectal cancer and colorectal adenoma, ICC and ECC, and breast, gastric, pancreatic, prostate, and esophageal cancers. However, the evidence for an association of NAFLD with various extrahepatic cancers remains insufficient. In addition, mechanistic details regarding the capacity for NAFLD to promote tumorigenesis remains unclear. Both of these aspects are important to consider in future studies.

## Supplementary information

**Additional file 1.**

**Additional file 2.**

## Data Availability

The datasets used and/or analyzed during the current study are available from the corresponding author upon reasonable request.

## Abbreviations

CI: Confidence interval
ECC: Extrahepatic cholangiocarcinoma
HR: Hazard ratio
ICC: Intrahepatic cholangiocarcinoma
ICD-9: International Classification of Diseases Ninth Revision
IRR: Incidence rate ratio
NAFLD: Nonalcoholic fatty liver disease
NASH: Nonalcoholic steatohepatitis
NOS: Newcastle-Ottawa Scale
OR: Odds ratio
PRISRMA: Preferred Reporting Items for Systematic Reviews and Meta-Analyses
