# Supplementary material for: Association between nonalcoholic fatty liver disease and extrahepatic cancers: a systematic review and meta-analysis
Source: Lipids Health Dis. 2020 May 31;19:118. doi: 10.1186/s12944-020-01288-6 (PMC7262754; doi:10.1186/s12944-020-01288-6)
Supplement: Supplementary file 1 — Additional file 1. [file 12944_2020_1288_MOESM1_ESM.docx]

**Supplementary Table 1.** Summary studies for the association between NAFLD and other cancers.

| **Cancer** | **Study** | **Sample**  **size** | **Country** | **NAFLD diagnosis** | **Patients source** | **Date** | **Study design** | **Cancer diagnosis** | **Adjusted confounding factors** | **Study quality** |
| --- | --- | --- | --- | --- | --- | --- | --- | --- | --- | --- |
| Gastric cancer | Allen et al. 2019 [21] | 30 | USA | ICD-9 | Community | 1997 - 2016 | Cohort | ICD-9 | NA | Good |
|  | Kim et al. 2018 [24] | NA | Korea | Ultrasonography | Community | 2004 - 2005 | Cohort | Pathology radiology | Demographic and metabolic factors | Good |
|  | Hamaguchi et al. 2019 [27] | 15926 | Japan | Ultrasonography | Community | 2004 - 2016 | Cohort | Endoscopy | Sex, age and lifestyle factors including smoking habits, alcoholic consumption and physical activities and diabetes. | Good |
| Pancreatic cancer | Allen et al. 2019 [21] | 70 | USA | ICD-9 | Community | 1997 - 2016 | Cohort | ICD-9 | NA | Good |
|  | Kim et al. 2018 [24] | NA | Korea | Ultrasonography | Community | 2004 - 2005 | Cohort | Pathology radiology | Demographic and metabolic factors | Good |
|  | Chang et al. 2018 [46] | 557 | China | CT | Hospital | 2009 - 2013 | Cohort | CT | NA | Good |
| Prostate cancer | Allen et al. 2019 [21] | 581 | USA | ICD-9 | Community | 1997 - 2016 | Cohort | ICD-9 | NA | Good |
|  | Cho et al.2019 [48] | 10million | Korea | hepatic steatosis index (HSI) | Community | 2009-2012 | Cohort | ICD-10 | age, smoking status, alcohol con-  sumption, exercise, income, diabetes, hypertension and dyslipidemia. | Good |
|  | Kim et al. 2018 [24] | NA | Korea | Ultrasonography | Community | 2004 - 2005 | Cohort | Pathology radiology | Demographic and metabolic factors | Good |
| Esophageal cancer | Allen et al. 2019 [21] | 29 | USA | ICD-9 | Community | 1997 - 2016 | Cohort | ICD-9 | NA | Good |
|  | Kim et al. 2018 [24] | NA | Korea | Ultrasonography | Community | 2004 - 2005 | Cohort | Pathology radiology | Demographic and metabolic factors | Good |

Abbreviation: ICD, International Classification of Diseases; CT, computed tomographic.
