# Supplementary material for: Association between nonalcoholic fatty liver disease and extrahepatic cancers: a systematic review and meta-analysis
Source: Lipids Health Dis. 2020 May 31;19:118. doi: 10.1186/s12944-020-01288-6 (PMC7262754; doi:10.1186/s12944-020-01288-6)
Supplement: Supplementary file 2 — Additional file 2. [file 12944_2020_1288_MOESM2_ESM.docx]

**
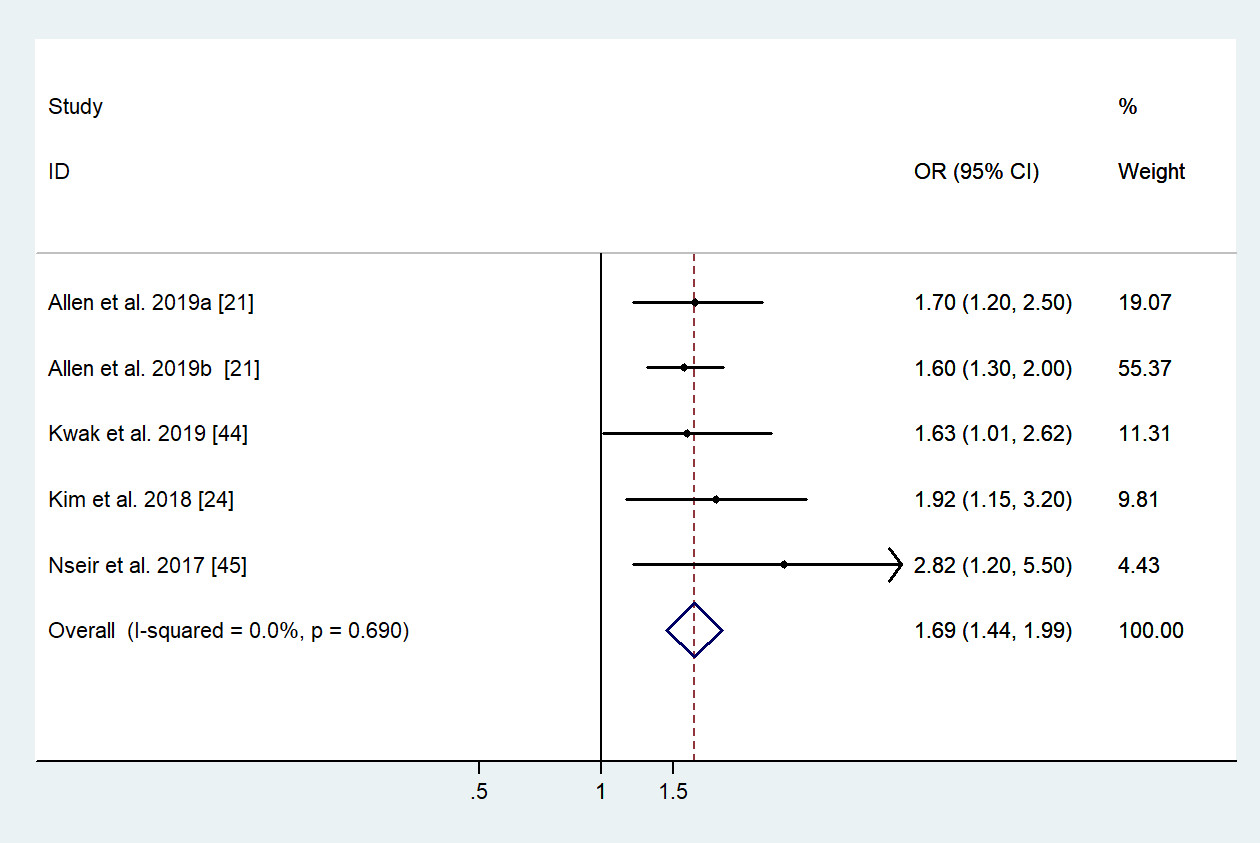
Supplementary Figure 1.** Forest plot of the meta-analysis of the association between NAFLD and breast cancer.
